# Understanding workforce sustainability through institutional theory: insights from Uzbekistan’s hotel industry

**DOI:** 10.3389/fsoc.2026.1930911

**Published:** 2026-09-10

**Authors:** H. Elif Kutlugun, Dilzodakhon Rakhimova, Berislav Andrlić

**Affiliations:** 1 Department of International Business, Business School, Central Asian University Tashkent, Uzbekistan; 2 Faculty of Tourism and Rural Development in Požega, University of Osijek, Osijek, Croatia

**Keywords:** hotel industry, human capital, institutional theory, SDGs, sustainability

## Abstract

This study explores hotel employees’ experiences in Uzbekistan using a phenomenological approach to understand how workplace conditions affect progress toward sustainable tourism guided by the primary research question: “What are the problems encountered in the hotel industry from employees” perspective in Uzbekistan? Through semi-structured interviews with 15 frontline employees, analyzed via an inductive-deductive thematic approach, the research captures employee views on motivation, turnover, skills, and daily challenges. Interpreted through the Sustainable Development Goals (SDGs) and Institutional Theory, the findings show that sustainability gaps arise from cross-pillar institutional misalignment rather than isolated managerial shortcomings. Weak enforcement of labor rules, industry norms that prioritize cost-minimization, and cultural expectations particularly around gender combine to produce wage inequity, limited career development, outdated training, infrastructure constraints, and neglected health and safety practices. Women additionally face restricted advancement due to unpaid care responsibilities and stigma attached to certain hospitality roles. These institutional pressures result in symbolic compliance with sustainability commitments instead of meaningful change. Ultimately, by highlighting the need for stronger labor enforcement, updated skills systems, improved infrastructure, gender-inclusive policies, and consistent safety practices, this study contributes theoretically by illustrating how macro-level institutional voids materialize in daily operations, and practically by offering practical strategies for policymakers and hoteliers to align institutional pillars and support a more equitable hotel sector in transition economies.

## 1 Introduction

The global tourism and hospitality industry has firmly established itself as a formidable engine of economic growth and job creation, with its burgeoning influence particularly evident across Asian markets, where substantial revenue increases contribute significantly to national GDPs (Darsana and Sudjana, 2022). In the context of emerging economies, tourism’s strategic importance transcends mere economic contributions, playing a vital role in fostering sustainable development and enhancing socio-economic well-being (Rai and Nayak, 2019).

In 2015, the United Nations launched the 2030 Agenda for Sustainable Development, outlining 17 Sustainable Development Goals (SDGs) aimed at eradicating poverty, reducing inequality, and addressing climate change (United Nations General Assembly, 2015). While only Goal 8 which is focused on decent work and economic growth explicitly references employment and workplace conditions, many of the other goals indirectly impact labor dynamics, especially within the tourism and hospitality sector. This places the concept of “decent work,” as defined by the International Labour Organization (ILO), at the heart of sustainability discussions in tourism. Decent work encompasses productive employment that delivers fair income, workplace security, social protection, and equal opportunities for all (International Labour Organization (ILO), 2012).

While sustainable tourism literature frequently emphasizes environmental conservation, the social dimension specifically workforce sustainability remains underexplored. In this study, workforce sustainability is defined as the provision of decent work, equitable compensation, structured career development, and safe working conditions that collectively foster long-term employee well-being and sectoral resilience (Baum et al., 2016a; Kramar, 2014). Despite its centrality to the United Nations Sustainable Development Goals (SDGs), particularly SDG 8 (Decent Work)—workforce sustainability is often overshadowed by rapid infrastructural expansion in emerging markets.

Uzbekistan, since its strategic pivot towards global engagement in 2016, has initiated sweeping reforms aimed at invigorating its tourism sector. The Presidential Decree on Tourism (2022) unequivocally articulates the nation’s ambitious vision: to generate an additional 3.5 million tourism jobs and to attract an unprecedented combined total of 21 million local and foreign tourists by 2027 (Development Strategy Center, Uzbekistan, 2022). While this expansion is undoubtedly promising for national prosperity, it simultaneously underscores the critical and immediate demand for a robust, highly skilled, and committed workforce (Rai and Nayak, 2019). However, the sustainability of this rapid growth model hinges on addressing fundamental challenges related to human resource management (Kramar, 2014). Indeed, issues such as the shortage of skilled labor and high employee turnover rates have the potential to jeopardize the long-term success of sectoral growth (Baum et al., 2016a, 2016b).

While this expansion is undoubtedly promising for national prosperity, it simultaneously underscores the critical and immediate demand for a robust, highly skilled, and committed workforce. However, the sustainability of this rapid growth model hinges on addressing fundamental challenges related to human resource management. Indeed, issues such as the shortage of skilled labor and high employee turnover rates have the potential to jeopardize the long-term success of sectoral growth.

The human-centered nature of hospitality makes the industry vulnerable to workforce-related challenges. Hotels face high employee turnover globally, but the impact is magnified in emerging markets due to a smaller pool of experienced professionals and fewer institutions offering modern, practice-oriented hospitality education (Baum, 2015). Each employee departure not only disrupts operational continuity but also results in the loss of tacit service knowledge that cannot be easily codified or replaced. This can lead to inconsistencies in service quality and a disruption of organizational culture, becoming one of the biggest obstacles to a sustainable growth model.

Uzbekistan’s hotel infrastructure has expanded rapidly, with official data indicating that the number of accommodation facilities increased more than sevenfold—from 767 in 2017 to 5,526 by the end of 2023—primarily concentrated in urban centers like Tashkent, Samarkand, and Bukhara (Daryo.uz, 2024; Hoteliers Association of Uzbekistan, 2022; National Statistics Committee of the Republic of Uzbekistan, 2024). This infrastructure development aligns with the government’s ambitious goal to raise hotel room capacity to 64,000 and attract 9 million foreign tourists by 2027 (Presidential Decree of the Republic of Uzbekistan, 2022). While these targets are promising, recent studies suggest that the speed of liberalization often outpaces the development of professional standards, leading to a “competency lag” in service-oriented sectors (Saidova, 2023).

While these targets are ambitious, recent studies on the ‘New Uzbekistan’ economic trajectory suggest that the speed of liberalization often outpaces the development of professional standards, leading to a ‘competency lag’ in service-oriented sectors (Saidova, 2023). Furthermore, the post-pandemic recovery has seen a global shift in employee expectations, where workers in transition economies increasingly prioritize job security and mental well-being over purely transactional employment (Baum et al., 2020; Seyitoğlu et al., 2023).

Nevertheless, it is not possible to say that this rapid growth in infrastructure aligns with a corresponding development in human capital. A significant gap in the knowledge, skills, number of workers, and loyalty required by the industry as well as significant difficulties in finding trained staff has been reported (Kutlugun et al., 2026), which is a problem that perpetuates a “low pay-low loyalty” cycle that affects employee satisfaction, service quality, and staff retention (Serhan et al., 2024). Moreover, public perception of tourism jobs is largely negative, which actively discourages the educated workforce from seeking employment in the sector. This has resulted in a continuous struggle for companies to hire and retain skilled personnel, leading to high employee turnover (Subramony, 2009; Van Iddekinge et al., 2017). This serious gap between infrastructural investment and human resource development depicts that although there are positive financial forecasts—with market revenue projected to have reached US $270.72 million in 2025 and to climb to US $438.31 million by 2030 (Statista, 2025), the long-term sustainability and quality of the industry is concerned, human capital deficiency in Uzbekistan, as well as rapid turnover in the tourism sector seems to be a serious issue that needs to be tackled immediately to reach the goals set for the future.

While existing literature has extensively documented operational HR challenges such as high turnover and skills shortages, there is a distinct lack of research analyzing these issues through a macro-theoretical lens in transition economies. Uzbekistan offers a highly theoretically interesting context: it is undergoing rapid state-driven tourism expansion, yet its labor market remains constrained by historical, post-Soviet bureaucratic legacies and deeply embedded socio-cultural norms. Therefore, this study adopts Institutional Theory to move beyond isolated managerial critiques and examine how regulative, normative, and cultural-cognitive pressures interact to create ‘institutional voids’ that hinder workforce sustainability. To address this gap, this study is guided by the following primary research question: How do institutional pressures and voids shape the lived experiences of hotel employees and affect workforce sustainability in Uzbekistan? Furthermore, the research pursues three specific objectives: (1) to identify the structural and interpersonal challenges faced by frontline hotel employees; (2) to examine how national institutional frameworks align or misalign with international SDG commitments; and (3) to propose actionable strategies for industry and policymakers. By doing so, this study makes a threefold contribution.

While existing literature has extensively documented operational HR challenges such as high turnover and skills shortages (Deery and Jago, 2015; Serhan et al., 2024), there is a distinct lack of research analyzing these issues through a macro-theoretical lens in transition economies. Uzbekistan offers a highly theoretically interesting context: it is undergoing rapid state-driven tourism expansion (Development Strategy Center, Uzbekistan, 2022), yet its labor market remains constrained by historical, post-Soviet bureaucratic legacies and deeply embedded socio-cultural norms (Seyitoğlu et al., 2023). Therefore, this study adopts Institutional Theory to move beyond isolated managerial critiques and examine how regulative, normative, and cultural-cognitive pressures interact to create “institutional voids”. Defined by Khanna and Palepu (1997) as the absence of specialized intermediaries, regulatory enforcement, and contract-enforcing mechanisms typical of advanced economies, institutional voids in Uzbekistan’s hotel sector manifest as an absence of credible labor enforcement mechanisms (regulative), a lack of modern skills-development institutions (normative), and gaps in social protection for vulnerable workers (cultural-cognitive).

Theoretically, it extends Institutional Theory by demonstrating how cross-pillar institutional misalignment produces symbolic compliance with SDGs in the hospitality sector. Contextually, it sheds light on the under-researched Central Asian transition economy of Uzbekistan. Practically, it provides a roadmap for policymakers and hoteliers to bridge the gap between rapid infrastructure growth and human capital development.

## 2 Theoretical framework

### 2.1 Sustainability and the SDGs

Sustainable development is classically defined as meeting the needs of the present without compromising the ability of future generations to meet their own needs (WCED, 1987). Within tourism and hospitality, the United Nations’ Sustainable Development Goals (SDGs) constitute a globally recognized framework for coupling economic vitality with social inclusion and environmental stewardship (United Nations General Assembly, 2015; United Nations World Tourism Organization (UNWTO), 2017). Although the sector has made notable operational strides adopting energy and water efficiency measures, reducing food waste, and experimenting with renewable technologies enduring sustainability transformation remains constrained by embedded organizational routines, labor market structures, and behavioral path dependencies globally (Gössling et al., 2015; Bohdanowicz, 2006). In order to understand why these constraints, persist despite widespread policy commitments and technological improvements, it is necessary to examine the institutional environments that shape organizational behavior in tourism and hospitality.

### 2.2 Institutional theory: origins and three pillars

Institutional Theory originated in the sociology of organizations to explain why organizations often adopt structures and practices not purely for operational efficiency, but to secure social legitimacy and survival (Meyer and Rowan, 1977; DiMaggio and Powell, 1983). Over time, the theory has been widely applied to the tourism and hospitality sector to understand how external pressures drive firms to adopt similar corporate social responsibility (CSR) and sustainability practices (Coles et al., 2013).

Institutional theory enriches sustainability analysis by explaining why organizations converge on particular practices, why these practices often look strikingly similar across firms and destinations, and why formal commitments do not always penetrate day-to-day routines. Tourism and hospitality firms face coercive pressures from laws, public regulation, multilateral standards, and certification schemes; normative pressures from professional associations, accreditation bodies, and occupational communities; and mimetic pressures that encourage imitation under uncertainty to protect legitimacy and reduce risk, together producing sectoral isomorphism in sustainability policy and reporting (DiMaggio and Powell, 1983; Scott, 2014). According to Scott (2014), these institutional forces operate through three foundational pillars: the regulative pillar (formal rules, laws, and sanctions), the normative pillar (industry expectations, values, and professional standards), and the cultural-cognitive pillar deeply embedded social beliefs and taken-for-granted cultural schemas In hospitality, these pressures are channelled through frameworks such as the Global Sustainable Tourism Council criteria, International Labour Organization conventions and the Decent Work Agenda, UN Guiding Principles on Business and Human Rights, and widely used reporting and management systems like GRI, ISO 14001/45001, and SA8000, which codify expectations for environmental and social performance and shape employment practices across global value chains (Global Sustainable Tourism Council, 2019; International Labour Organization (ILO), 2012; Office of the High Commissioner for Human Rights (OHCHR), 2011; Matten and Moon, 2008). The result is visible convergence in policies codes of conduct, supplier standards, human-rights commitments, diversity pledges, and sustainability reports even when firms compete in different markets and institutional contexts (Aguilera et al., 2007; Coles et al., 2013). Crucially, these three pillars are not separate; they interact and reinforce one another. For example, weak regulative enforcement of labor laws enables cost-minimizing industry norms. These norms, in turn, reinforce deeply rooted cultural-cognitive beliefs that naturalize poor working conditions or gendered occupational segregation, creating a structural barrier to sustainability.

### 2.3 Decoupling and symbolic compliance

Yet formal adoption can diverge from lived practice. It is critical to distinguish between two related concepts: decoupling and symbolic compliance. Decoupling typically refers to an unintentional or structural gap between formal policies and operational practices, often caused by resource constraints or institutional misalignment. In contrast, symbolic compliance suggests an intentional, strategic signaling by firms to gain legitimacy without taking substantive action (Meyer and Rowan, 1977). In emerging markets, both mechanisms can occur, particularly where short-term incentives dominate, informality is prevalent, monitoring capacity is thin, and enforcement is weak (North, 1990).

Yet formal adoption can be decoupled from lived practice. Decoupling arises when organizations ceremonially adopt structural policies, standards, and reports to signal conformity while insulating core operations from costly change, particularly where short-term incentives dominate, informality is prevalent, monitoring capacity is thin, and enforcement is weak (Meyer and Rowan, 1977; North, 1990). Empirical studies document how firms publicly embrace sustainability while selectively implementing or symbolically complying, for instance by emphasizing easy-to-measure KPIs and marketing narratives while neglecting difficult labor issues such as subcontracted housekeeping, seasonal layoffs, or migrant worker protections (Delmas and Toffel, 2008; Bromley and Powell, 2012). In tourism, rhetorical commitments and glossy reports may exceed operational reality when supply chains are fragmented and destination governance is uneven; sustainability communication can become a form of impression management unless backed by credible auditing, worker voice, and incentives aligned to operational change (Font and McCabe, 2017; Coles et al., 2013). Research on stakeholder pressures shows that under competing demands, firms may “selectively couple,” implementing what is visible to external audiences and postponing deeper changes to labor processes and supervisory practices that affect daily work experience (Crilly et al., 2012; Boiral, 2013).

### 2.4 Sustainable HRM and the SDGs

Tourism employment constitutes a critical pillar of sustainable development because it can generate broad-based livelihoods, catalyze local enterprise, and connect marginalized groups to formal labor markets, while also carrying risks of precarity that must be institutionally governed. The sector’s labor intensity, low formal entry barriers, and geographic dispersion create opportunities for women, youth, migrants, and rural populations, linking directly to SDG 8 on decent work and inclusive growth and indirectly to SDG 1 on poverty reduction and SDG 10 on reduced inequalities (United Nations World Tourism Organization (UNWTO), 2018; International Labour Organization (ILO), 2012). However, the same features can produce seasonal contracts, irregular hours, and subcontracting that externalize risk onto workers; without robust institutions and sustainable HRM, tourism jobs may be plentiful but precarious, undermining both equity and long-term service quality (Baum, 2015; Baum et al., 2016b). Institutionalized standards collective agreements, public procurement clauses, destination-level certification, and social dialogue are thus not peripheral but foundational mechanisms for transforming tourism’s quantitative job creation into qualitatively decent and sustainable employment (Global Sustainable Tourism Council, 2019; Scott, 2014).

Tourism jobs contribute to the Sustainable Development Goals through the protection and fulfillment of human rights in the workplace and along supply chains. Applying the UN Guiding Principles’ pillars state duty to protect, corporate responsibility to respect, and access to remedy requires robust due diligence on risks such as excessive working hours, discrimination and harassment, unsafe working conditions, wage theft, forced labor in outsourced services, and restrictions on freedom of association, with particular vigilance for women workers, migrants, and Indigenous communities (Office of the High Commissioner for Human Rights (OHCHR), 2011; Jamali, 2010). Embedding the ILO’s core labor standards and Decent Work dimensions employment creation, rights at work, social protection, and social dialogue into hotel operations, tour operations, and platform-mediated services links directly to SDG targets 8.5 (full and productive employment and equal pay), 8.7 (eradication of forced labor), and 5.2/5.5 (eliminating violence and ensuring women’s full participation) (International Labour Organization (ILO), 2012; Matten and Moon, 2008). When firms move beyond policy statements to operational practices transparent recruitment (no fees to workers), safe accommodation for staff, grievance mechanisms, joint worker-management committees, and remediation pathways human-rights due diligence becomes a tangible driver of development outcomes in destinations (Aguilera et al., 2007; Crilly et al., 2012).

Employee retention is both a social outcome and a strategic lever for sustainability in tourism. Chronic turnover driven by seasonality, variable hours, limited advancement, and low wages erodes service quality, inflates recruitment costs, and dissipates firm-specific human capital; conversely, equitable pay structures, predictable scheduling, participatory work design, and visible internal mobility reduce quit rates and strengthen organizational commitment (Deery and Jago, 2015; Hinkin and Tracey, 2000). A sustainable HRM bundle linking wellbeing (e.g., safe workloads and mental health support), development (e.g., paid training and clear career ladders), and justice (e.g., transparent appraisal and non-discrimination) is associated with lower turnover intentions and higher service performance, reinforcing SDG 8’s emphasis on productive employment and decent work (Jiang et al., 2012; Ehnert, 2009). Institutional context matters: where sectoral bargaining, destination-level standards, and credible enforcement exist, retention-enhancing practices diffuse faster and avoid a “race to the bottom” in labor standards; where governance is weak, mimetic adoption without robust implementation sustains high churn and precariousness (Paauwe and Boselie, 2003; Scott, 2014).

Sustainability and institutional theory converge on the need to embed sustainable HRM into the rules, routines, and incentives that structure tourism employment, thereby minimizing decoupling. Organizationally, this requires integrating social objectives into core strategy and control systems tying executive bonuses and property-level dashboards to leading indicators such as training hours, internal promotion rates, injury rates, and retention of target groups; aligning procurement with labor standards; and ensuring credible third-party audits with worker participation (Kramar, 2014; Delmas and Toffel, 2008). Institutionally, it involves leveraging multi-stakeholder platforms and destination stewardship to co-produce standards and monitoring, using recognized frameworks GSTC criteria for destinations and businesses, ILO conventions, and UNGPs as the common grammar for expectations, measurement, and remedy across owners, brands, franchisees, and contractors (Global Sustainable Tourism Council, 2019; Office of the High Commissioner for Human Rights (OHCHR), 2011). Where organizational fields are characterized by conflicting logics cost-minimizing franchising versus quality-assuring brand management managers can “re-couple” by creating hybrid practices that satisfy both legitimacy and efficacy, for example by standardizing labor-rights due diligence in franchise agreements and tying brand compliance audits to HRM outcomes, thus moving from symbolic to substantive conformity (Pache and Santos, 2010; Bromley and Powell, 2012).

Taken together, this framework explains why tourism firms tend to look similar in their sustainability talk, why their walk often lags, and how to close the gap by institutionalizing human rights, capability development, and retention-oriented HRM as non-negotiable components of business performance. Coercive, normative, and mimetic pressures are not merely background conditions: when coupled to credible enforcement, stakeholder voice, and internal incentives, they become the engines that translate sustainability’s aspirations into the everyday practices that constitute decent, developmental, and durable work in hospitality thereby advancing the SDGs through substantive, embedded practices rather than merely superficial compliance (DiMaggio and Powell, 1983; International Labour Organization (ILO), 2012; United Nations World Tourism Organization (UNWTO), 2018; Scott, 2014).

### 2.5 Tourism employment and institutional logics in sustainable development

Since the mid-1990s, sustainability has moved to the center of tourism discourse. However, workforce considerations—decent work, structured career progression, and social inclusion—have often been treated as peripheral, despite their centrality to the UN’s Sustainable Development Goals (SDGs 1, 3, 4, 5, 8, and 10) (Miller et al., 2010; Global Sustainable Tourism Council, 2019). Institutional mechanisms clarify the persistence of this gap. Normative pressures elevate sustainability narratives, yet HR systems often remain configured for short-term efficiencies, producing decoupling between sustainability disclosures and everyday labor conditions (Scott, 2014; Meyer and Rowan, 1977). Coercive pressures anchored in national labor law aim to embed decent work, but enforcement remains uneven.

To address these tensions, Sustainable Human Resource Management (HRM) prioritizes long-term workforce well-being and capability-building rather than short-term cost containment (Ehnert, 2009). In the context of Uzbekistan, these institutional dynamics are uniquely shaped by its status as a rapid-transition economy operating within ‘institutional voids’ (Khanna and Palepu, 1997). Hotels face a coercive push from the state to modernize rapidly, juxtaposed with normative industry practices of cost-minimization, and deeply rooted cultural-cognitive legacies related to gender from the post-Seyitoğlu era. It is within these specific local voids that symbolic compliance with global sustainability standards occurs. Workforce sustainability ultimately hinges on the alignment of institutional pressures with substantive HR architectures that build human potential, protect rights, and enable long-term employability.

Hotels face a coercive push from the state to modernize rapidly (Saidova, 2023), juxtaposed with normative industry practices of cost-minimization (Baum, 2015), and deeply rooted cultural-cognitive legacies related to gender from the post-Soviet era (Seyidov et al., 2023). It is within these specific local voids that symbolic compliance with global sustainability standards occurs (Khanna and Palepu, 1997; Bromley and Powell, 2012). Workforce sustainability ultimately hinges on the alignment of institutional pressures with substantive HR architectures that build human potential, protect rights, and enable long-term employability (Kramar, 2014; Scott, 2014).

## 3 Materials and methods

### 3.1 Research design

To explore the lived experiences and perspectives of employees in Uzbekistan’s hotel sector, this study employed a phenomenological methodology within a qualitative research framework (Denzin, 1997; Marshall and Rossman, 2012). This approach was selected for its emphasis on capturing participants’ subjective realities, offering nuanced understanding of complex issues such as employee motivation, staff turnover, and career development-dimensions that are often inadequately addressed through quantitative analysis alone. This study aims for analytical rather than statistical generalization (Yin, 2014), using empirical evidence to refine and advance theoretical perspectives on institutional voids in emerging markets. Guided by a phenomenological orientation, the research privileges the depth and nuance of lived experience over wide-scale sampling. Fifteen semi-structured interviews were carried out in three hotels located in Tashkent—Uzbekistan’s administrative hub and the center of national tourism policy reforms.

A purposive sampling strategy was employed to select 15 frontline employees. In phenomenological research, samples of this size are standard as the objective is to capture the depth of lived experience rather than statistical generalizability (Creswell and Poth, 2018). The sample size was determined based on the principle of thematic saturation. Saturation was actively monitored during data collection by comparing new interview transcripts against the developing coding framework; specifically, we tracked when no new codes emerged from the data. By the thirteenth interview, recurring themes had stabilized and informational redundancy was achieved, meaning no substantially new structural themes or institutional challenges emerged from the employee narratives. The sample intentionally included participants with varying tenure, including those with under 2 years of experience. While junior employees may have less historical exposure to long-term institutional constraints, they provide crucial, immediate insights into current onboarding gaps and the transition from formal education to actual industry practices.

The final two interviews were conducted specifically to confirm this thematic stability, verifying that the sample size was sufficient to capture the depth of lived experiences and institutional constraints within the scope of 4-star properties.

The selected hotels function as institutional interface organizations positioned at the crossroads of labor regulation, industry standards, tourism governance, and cultural norms. This makes them strategic sites for observing how broader institutional inconsistencies materialize in everyday organizational dynamics. Accordingly, the study seeks to generate theoretical insights into the mechanisms that produce institutional voids, rather than pursue numerical representativeness.

Data collection was conducted through in depth, semi-structured interviews, allowing participants to articulate their personal narratives and workplace experience in their own terms. This method facilitated the generation of rich, contextually embedded insights that contribute to more holistic understanding of human dynamics within the hospitality sector.

### 3.2 Participants of the study

Phase 1 involved a pilot phase with three hotel managers to obtain strategic insights into organizational structures, evaluate the context, and refine the interview instrument. Because this was a pilot to finalize the questions, the responses of these managers are not included in the main dataset. Furthermore, excluding managerial data from the final analysis was a deliberate methodological choice to center the lived, everyday realities of frontline workers, who directly experience the consequences of institutional voids. While managers often articulate formal policies and strategic intent, frontline employees provide the critical perspective needed to identify where these policies diverge into symbolic compliance.

Phase 2 comprised the primary data collection, where semi-structured interviews were conducted with frontline employees representing five distinct operational departments: front office, housekeeping, food and beverage service, spa & fitness, and kitchen. A total of fifteen staff members were selected from three hotels located in Tashkent, Uzbekistan’s capital and a key tourism destination. Starting data collection in Tashkent was a deliberate methodological choice. As the administrative and economic hub, Tashkent serves as the center of national tourism policy reforms and possesses a relatively developed hospitality infrastructure. Exploring this context provides a valuable baseline for understanding how institutional pressures manifest in the most formalized segment of the country’s hotel sector. While it is possible that institutional voids—such as regulatory gaps or infrastructural challenges—might be experienced differently, or perhaps more acutely, in secondary tourism destinations like Samarkand, Bukhara, or Khiva due to their distinct local contexts, establishing this initial understanding in the capital is a necessary first step. This approach grounds the research, laying the foundation for future comparative studies to explore regional variations across Uzbekistan’s wider tourism landscape.

The sample specifically targeted mid-sized, 4-star hotels (averaging approximately 50 rooms). This deliberate choice was made because 4-star properties represent a critical intersection: they attempt to formalize HR structures but remain heavily exposed to local institutional pressures, unlike international 5-star mega-chains whose imported corporate standards often insulate them from local institutional voids. All participants had a minimum of 1 year of professional experience in the hotel industry, ensuring they possessed sufficient familiarity with workplace realities and service expectations. This multi-tiered sampling strategy enabled the study to capture a diverse range of perspectives across hierarchical levels, thereby enriching the overall analysis of workforce experiences within the hospitality domain. Table 1 presents detailed demographic and professional information about the interview participants.

**Table 1:** The codes of and demographic information of the participants.

Participant	Gender	Age	Department	Role	Experience in hotel industry	Hotel status
Participant (P1)	Female	28	F&B	Supervisor	11 years	4-star
Participant (P2)	Female	42	Housekeeping	Assistant	16 years	4-star
Participant (P3)	Male	21	Front office	Concierge	5 month	4-star
Participant (P4)	Male	22	F&B	Waiter	2 years	4-star
Participant (P5)	Male	20	Front office	Reception	2 years	4-star
Participant (P6)	Female	20	Housekeeping	Maid	1,5 years	4-star
Participant (P7)	Male	18	F&B	Waiter	1,5 years	4-star
Participant (P8)	Female	32	Spa&Fitness	Manager	6 years	4-star
Participant (P9)	Male	21	Kitchen	Junior chef	1 year	4-star
Participant (P10)	Female	30	Housekeeping	Laundry attendant	1 year	4-star
Participant (P11)	Female	25	Housekeeping	Assistant	1 year	4-star
Participant (P12)	Male	23	Front office	Reception	1,5 years	4-star
Participant (P13)	Male	26	Front office	Concierge	8 months	4-star
Participant (P14)	Female	30	Spa&Fitness	Spa Therapist	2,5 years	4-star
Participant (P15)	Male	34	F&B	Supervisor	5 years	4-star

### 3.3 Data collection instrument

This study is guided by the central research question: “What are the problems encountered in the hotel industry from employees’ perspective in Uzbekistan?”

The objective is to gain a nuanced understanding of the challenges hotel employees face in their daily work, professional development and interactions within the broader tourism ecosystem. The inquiry focuses on both structural and interpersonal factors that affect employee satisfaction, retention and service delivery.

To address this question, a semi-structured interview guide was carefully constructed following an extensive review of scholarly literature on hospitality employment, tourism development and labor conditions in emerging markets. The guide was specifically adapted to reflect the socio-cultural and economic realities of Uzbekistan’s hotel sector.

Before implementation the interview guide underwent a pilot phase involving three hotel managers who were not included in the main study. Their insights were instrumental in refining the phasing, order and thematic coherence of the questions, ensuring that the instrument was both clear and contextually appropriate.

The interview topics covered a wide range of areas, beginning with participants’ entry into the tourism industry and their motivations for choosing this career path. Questions also explored their educational and training experiences, the variety of hotel departments they had worked in and their views on the effectiveness of formal tourism education in preparing them for practical roles. Additional themes included perceptions of Uzbekistan’s tourism landscape, general and employee-specific challenges within the hotel sector and factors contributing to staff turnover. Participants were also asked about their long-term career aspirations, the societal value they associate with their work and how their families perceive their professional roles.

To ensure linguistic inclusivity and precision the interview guide was translated into Uzbek and Russian languages. These translations were reviewed by qualified linguists and the reliability of the instrument was verified using the back translation method. This process ensured that the meaning of each question remained consistent across languages and that participants could engage with the interview in their preferred language without loss of clarity or intent.

### 3.4 Data collection procedure

The qualitative data collection was carried out through individual, in-person interviews with hotel staff across three different hotels in Tashkent. Each session was arranged in advance to ensure convenience for participants and to create a comfortable setting conducive to open dialogue. Interviews were conducted within the hotel premises, in quiet and private spaces that allowed for uninterrupted conversation and confidentiality.

Before each interview began, participants were fully briefed on the purpose and scope of the study. They were informed of their rights, including the option to withdraw at any stage without explanation, and the steps taken to protect their privacy. Both verbal and written consent were obtained for participation and audio recording, ensuring ethical standards were upheld throughout the process.

The interviews followed a semi-structured format, guided by a set of nine open-ended questions. This approach allowed for consistency across sessions while also providing the flexibility to explore individual experiences in greater depth. Each interview lasted between 30 and 45 min, depending on the participant’s responses and engagement.

All conversations were audio-recorded with permission and later transcribed verbatim to preserve the integrity of the data. These transcripts formed the basis for subsequent qualitative analysis, enabling the researcher to identify recurring themes, patterns, and insights relevant to employment in the hospitality and tourism sector.

### 3.5 Data analysis

Interview audio recordings were transcribed and the data was analyzed using a structured content analysis approach, guided by the four-stage framework outlined by Marshall and Rossman (2012). Specifically, a thematic analysis utilizing an inductive-deductive approach was applied. The deductive phase was guided by *a priori* concepts from Institutional Theory and the SDGs, while the inductive phase allowed new themes to emerge naturally from the workers’ lived experiences. Due to the manageable volume of the dataset, coding was conducted manually using Microsoft Word highlighting and marginal notes.

The coding framework was developed iteratively. *A priori* codes were derived from Institutional Theory (e.g., regulative pressures, normative constraints, cultural-cognitive schemas) and the SDGs (e.g., decent work, gender equity). Inductive codes (e.g., wage compression, outdated curricula, infrastructure deficits) emerged directly from the participant narratives. To ensure inter-rater reliability and prevent researcher bias, two researchers independently coded the initial transcripts. Discrepancies were discussed during peer-debriefing sessions until a consensus was reached, after which a unified coding framework was applied to the full dataset. This process involved organizing the transcripts, identifying recurring themes and patterns, validating those themes against the full dataset, and considering alternative interpretations to ensure analytical depth. Participant identities were anonymized using coded identifiers (e.g., P1, P2, P3), maintaining confidentiality throughout the analysis (see Table 1).

To ensure inter-rater reliability and prevent researcher bias, two researchers independently coded the initial transcripts. Discrepancies were discussed during peer-debriefing sessions until a consensus was reached, after which a unified coding framework was applied to the full dataset. This process involved organizing the transcripts, identifying recurring themes and patterns, validating those themes against the full dataset, and considering alternative interpretations to ensure analytical depth. To strengthen the reliability of the findings, researchers independently coded the transcripts. Their results were then compared and reconciled to ensure consistency. Participant identities were anonymized using coded identifiers (e.g., P1, P2, P3), maintaining confidentiality throughout the analysis (see Table 1). Upon completion of the data analysis, the subsequent section of the paper will examine emergent themes and critically engage with particularly salient participant responses.

## 4 Results

The findings of this study reveal that workforce sustainability challenges in Uzbekistan’s hotel sector are deeply systemic. Viewed through the lens of institutional theory, the interview data illustrates how the daily workplace experiences of employees are shaped by the complex interplay of regulative, normative, and cultural-cognitive structures (Scott, 2014). Rather than attributing sustainability gaps to isolated managerial shortcomings, the findings underscore systemic institutional constraints that hinder the effective integration of sustainability discourse and Sustainable Development Goals (SDGs) into routine organizational practices. Qualitative evidence reveals that sustainability in hospitality is not merely an operational or technical concern but a deeply institutionalized phenomenon, embedded in wage-setting practices, educational systems, investment patterns, gender norms, and occupational health standards.

The discussion aligns these findings with SDGs 1, 3, 4, 5, 8, and 9, illustrating how institutional misalignments compromise the social, economic, and human dimensions of sustainability in an emerging tourism context.

### 4.1 Structural fragility of hotel employment (SDG 1 and SDG 8)

A dominant theme emerging from this study is the structural fragility of hotel employment, characterized by high staff turnover, wage compression, and limited career progression. From an institutional perspective, these conditions reflect a weak regulative framework, where labor laws and wage protections exist formally but lack robust enforcement, particularly in service sectors marked by high labor flexibility and minimal unionization (International Labour Organization (ILO), 2023a,b; Scott, 2014). Moreover, recent analysis of Central Asian labor markets suggests that the ‘informality trap’ where workers are hired without clear career pathways remains a primary barrier to professionalizing the hospitality sector, leading to a permanent state of precariousness for frontline staff (Yunusov and Rakhmanov, 2024).

When asked about the primary factors driving staff turnover in their departments, participants across hierarchical levels consistently identified low and inequitable wages as the primary catalyst for employee attrition. This finding is strongly supported by frontline accounts. As P5 explained, “In 5 months, more than five people changed… just one problem, not enough salary,” highlighting the direct relationship between low wages and turnover. For instance, P1, a food and beverage supervisor with over 10 years of experience, emphasized the demoralizing impact of salary compression, noting that newly recruited staff often earn salaries comparable to long-serving employees. This perception of inequity is echoed by employees themselves. As P1 noted, “If I’ve been working here for 10 years I get the same salary as those who started working recently,” illustrating how wage compression undermines fairness. Such practices undermine normative expectations of fairness and meritocracy, which institutional theory regards as essential for organizational legitimacy and employee compliance (Suchman, 1995).

One supervisor further articulated the systemic nature of turnover:

*“The moment a new hotel opens, it is highly likely that we will lose some of our good employees. They leave us even for a slightly better salary or for a title… all that training is wasted and we have to start all over again. It is not really sustainable.”*

This account illustrates how short-term competitive labor practices have become institutionalized, creating a self-reinforcing cycle of instability. Employees also recognize the operational consequences of this instability. As P5 stated, “Finding the new employee is easy, but educating them is hard,” indicating how constant turnover creates ongoing training burdens. Rather than prioritizing retention through equitable compensation and career development, organizations normalize turnover as an inevitable sectoral feature. According to North (1990), such path-dependent practices persist when institutions fail to internalize long-term efficiency considerations.

The most acute economic vulnerability was observed among frontline employees-particularly kitchen staff, room-service workers, and security personnel who perform physically demanding tasks while receiving the lowest wages. One interview with a young kitchen worker revealed the intergenerational dimensions of economic insecurity: he temporarily replaced his ill mother at work to prevent her salary from being reduced, as her income was essential for family survival. This narrative demonstrates how hotel employment, rather than alleviating poverty, can perpetuate household-level precarity, contradicting the core premise of SDG 1.

From a sustainability standpoint, this finding is critical. Tourism is often promoted as a poverty-reduction strategy through job creation; however, when employment fails to provide a living wage, it results in what Baum et al. (2016b) term “working poverty” a condition where formal employment does not guarantee economic security. This condition is clearly reflected in employee experiences. As P9 explained, “They give us a lot of work, but the pay doesn’t match the amount of work we do,” demonstrating the imbalance between labor intensity and compensation. This lived reality directly contravenes the core tenets of SDG 8 (Decent Work and Economic Growth), specifically Target 8.5, which advocates for full and productive employment and equal pay for work of equal value.

Trust erosion further compounds this issue. A restaurant manager remarked:

*“There were a lot of promises, but not fulfilled. That’s why a lot of people leave.”*

Unfulfilled promises regarding promotions, wage increases, and working conditions undermine organizational legitimacy, a critical institutional resource for stability and employee commitment (Suchman, 1995). When managerial commitments are perceived as symbolic rather than substantive, employee exit becomes a rational and socially accepted response. This erosion of trust is evident in employee narratives. As P5 noted, “There were promises, but not fulfilled. That’s why a lot of people leave,” highlighting how unmet expectations weaken organizational legitimacy.

Collectively, these participant narratives suggest that from the perspective of frontline workers, the current state of wage regulation and career progression limits the industry’s ability to meaningfully deliver on the micro-level promises of SDG 1 and SDG 8. Economic growth without decent work risks reinforcing inequality rather than reducing it.

### 4.2 Mismatch between theory and practice (SDG 4)

Another significant barrier to sustainability identified in this study is the persistent disconnect between formal tourism education and the practical requirements of the hotel industry. Interviewees consistently described tourism curricula as outdated, referring to them as based on an “old Soviet standard program.” Employees consistently emphasized the limited applicability of formal education. As P1 stated, “There is little in theory, more through practice… I learned through practice,” indicating that experiential learning dominates. This reflects institutional inertia within educational systems, where historical legitimacy and bureaucratic continuity overshadow responsiveness to market and societal change (DiMaggio and Powell, 1983; Scott, 2014).

Most participants reported acquiring relevant skills almost exclusively through on-the-job training, regardless of whether they had completed tourism-related education. This reliance on informal learning is further confirmed by P6, who explained, “I learned everything from scratch here… I learned everything while working,” highlighting the marginal role of formal education. Even graduates of tourism high schools and universities emphasized that formal education failed to prepare them for real-world hospitality operations. This reliance on informal learning is further confirmed by P6, who explained, “I learned everything from scratch here… I learned everything while working,” highlighting the marginal role of formal education. This profound disconnect exposes a systemic failure to achieve SDG 4 (Quality Education), particularly Target 4.4, which emphasizes the need to substantially increase the number of youth and adults who have relevant skills for employment and decent jobs.

In response to questions regarding how well formal tourism education prepared them for their current roles, P3, a long-serving room service supervisor, described the resulting workplace tensions:

*“Old employees and their ways of doing things are different from the new ones… young employees bring knowledge that does not really work out in real life. It is just going by the book but not knowing the practice.”*

This statement underscores how symbolic knowledge acquired through formal education often lacks contextual relevance. Institutional theory explains this as a form of decoupling, where educational credentials signal competence without necessarily producing functional capability (Meyer and Rowan, 1977). Even those without formal tourism education reported similar experiences. As P4 noted, “I didn’t have any special training… they taught me everything here,” reinforcing the gap between education and practice. Consequently, hotels are compelled to compensate through costly and time-intensive on the job training, which further exacerbates turnover and inefficiency.

The implications for sustainability are profound. Skills mismatches undermine service quality, reduce productivity, and hinder innovation, directly affecting the sector’s competitiveness. Moreover, they weaken the social sustainability of tourism by limiting employees’ professional development and career mobility. Without meaningful reform in tourism education and stronger collaboration between educational institutions and industry stakeholders, SDG 4 remains aspirational rather than achievable.

### 4.3 Infrastructural deficits (SDG 9)

Infrastructural deficits surfaced as a further decisive institutional constraint. Participants consistently characterized a significant share of hotels as dated, insufficiently equipped, and falling short of international benchmarks. Operational limitations further illustrate these infrastructural gaps. As P4 reported, “We couldn’t accept card payments at the table… guests had to go to the cashier,” reflecting outdated service systems. As one informant (P5) observed:

*“Uzbekistan’s hotels need a lot of investment to be renovated.”*

This physical degradation is compounded by operational bottlenecks that directly impact daily work. As P2, a housekeeping assistant, explained:

*“Our service elevator breaks down frequently, which means we have to carry heavy laundry bags up the stairs. It slows everything down and exhausts the staff.”*

Similarly, front office employees bear the brunt of aging facilities when facing guests. P12 noted:

*“The air conditioning system is very old. During the summer, it cannot cool the rooms properly, and guests constantly complain to the reception, but we cannot do anything because the whole building’s system needs replacing.”*

These specific infrastructure limitations directly obstruct progress toward SDG Target 9.4, which calls for upgrading infrastructure and retrofitting industries to make them sustainable. Beyond the material degradation of facilities, respondents underscored the lack of environmental integration, particularly in urban properties where hotels are conceived as standalone edifices detached from natural elements.

*“We need to bring nature inside the hotels… especially in Tashkent, hotels are just buildings, isolated from nature.”*

These insights suggest a nascent reorientation in sectoral norms toward sustainability-focused expectations, even as prevailing infrastructure remains misaligned with these values. At a broader level, P2 highlighted structural limitations, noting that “There aren’t many 5-star hotels… things like transport and facilities are not fully developed,” indicating gaps in tourism infrastructure. From an institutional theory perspective, such dynamics indicate an emergent phase of institutional transformation wherein evolving normative preferences challenge entrenched material arrangements (Scott, 2014).

For the interviewed employees, aging infrastructure is not merely a macroeconomic issue; it directly constrains their daily operational performance and impinges on their workplace well-being. Conversely, deliberate investment in biophilic design, green spaces, and resource-efficient systems can produce multivalent gains, enhancing staff health, guest experience, and environmental outcomes concomitantly. Accordingly, infrastructural modernization should be conceived not as a solely technical endeavor but as an institutional project necessitating synchronized adjustments across regulatory regimes, financing instruments, and cultural acceptance of sustainability principles.

### 4.4 Occupational health, safety, and institutional neglect (SDG 3)

Issues of workplace health and safety, particularly in back-of-house departments expose a further layer of institutional shortfall. When asked about what kind of problems they face while doing their jobs, A laundry worker reported:

*We don’t have masks and gloves for our job. We work with a lot of dust and chemicals.*

This testimony illuminates the institutional invisibility of lower-status roles, wherein exposure to occupational hazards is normalized and preventive provisions routinely overlooked. This lack of protection is clearly articulated by employees. As P10 stated, “We need masks… gloves… we inhale a lot of dust,” highlighting inadequate safety measures. This lack of protective equipment translates directly into physical harm. P6, a housekeeping maid, detailed the daily reality of chemical exposure: “We use strong cleaning liquids for the bathrooms every day, but nobody explains the risks. By the end of the shift, my throat hurts and my eyes are red, but we just have to keep working.”

Similarly, routine physical injuries in back-of-house operations are often dismissed. P9, a junior chef, shared his experience with workplace hazards: “In the kitchen, the floors are often slippery and we get minor burns or cuts from rushing with old equipment, but there is no real focus on our safety or even a proper first aid kit available.” Although international labor standards codify the right to safe working conditions, deficient enforcement in practice permits such risks to persist (International Labour Organization (ILO), 2023a,b).

This lack of protection is clearly articulated by employees. As P10 stated, “We need masks… gloves… we inhale a lot of dust,” highlighting inadequate safety measures. Although international labor standards codify the right to safe working conditions, deficient enforcement in practice permits such risks to persist (International Labour Organization (ILO), 2023a,b). Moreover, attempts to raise these concerns often go unaddressed. As P10 explained, “If we point out issues, they acknowledge it briefly and then do nothing… our words are not taken into account,” demonstrating weak institutional responsiveness. Viewed through an institutional lens, these conditions denote both weak regulative capacity and a normative tolerance for risk among marginalized occupational groups. For these workers, the absence of basic protective equipment represents a lived experience of institutional neglect. The normalization of such hazards stands in stark opposition to SDG 3 (Good Health and Well-being), specifically Target 3.9, which aims to substantially reduce illnesses from hazardous chemicals, as well as SDG 8.8, which mandates the protection of labor rights and the promotion of safe and secure working environments.

### 4.5 Gender equality and cultural-cognitive constraints (SDG 5)

Gendered inequities appear as deeply embedded institutional phenomena. While talking about the challenges they faced, female participants reported that professional obligations are compounded by cultural expectations assigning women primary responsibility for unpaid reproductive labor, including childcare and eldercare. Male respondents frequently depicted hotel work as intrinsically ill-suited to women, these perceptions are reinforced by structural constraints. As P4 noted, “If the work starts at 4 PM and ends at 1 AM… it may not be suitable for women,” indicating how working hours disadvantage female employees.

Notably, none of the women interviewed attributed their difficulties to incapacity. Rather, they emphasized the absence of supportive social and institutional structures. As one participant explained:

*Nobody supports me and nobody thinks that I have a career worth keeping.*

This distinction is pivotal: barriers arise not from deficits in competence, but from institutionalized gender norms that devalue women’s paid employment and constrain their occupational participation. Cultural expectations further limit women’s participation. As P4 explained, “Women may not get permission from their family members to work these hours,” illustrating how social norms shape employment opportunities.

Institutional theory situates these norms within the cultural-cognitive pillar, which often resists change more stubbornly than formal regulation (Acker, 2006). Additionally, cultural stigmas regarding women’s employment in specific hotel functions such as spa services further impede labor market inclusion. As P14, a female spa therapist, shared: “When people hear I work in a hotel spa, they sometimes make inappropriate assumptions. It makes it very hard to feel proud of my profession.” Male participants also echoed these deeply ingrained societal views regarding hospitality work. P15, a male food and beverage supervisor, remarked: “In our culture, it is generally believed that a respectable woman should be at home with her family in the evenings, not serving guests late at night in a hotel.” Such structural constraints are a direct impediment to SDG 5 (Gender Equality).

Without institutional interventions to recognize and support unpaid care work (Target 5.4) and ensure women’s full and effective participation and equal opportunities for leadership (Target 5.5), the sector will continue to reproduce gendered disparities, ultimately attenuating progress toward SDG 8 by restricting women’s access to secure, dignified work and career progression.

## 5 Discussion

When the findings of this study are analyzed, it has been observed that there exists multifaceted barriers to sustainable tourism development in Uzbekistan. These insights are consistent with broader literature on the challenges faced by emerging tourism markets, where sustainability efforts often struggle with implementation despite the fact that they are being embedded in strategic plans (Vrana, 2023). This study demonstrates that sustainability shortfalls in Uzbekistan’s hotel sector stem from cross-pillar institutional misalignment rather than isolated managerial or technical deficits.

Following institutional theory, organizational practice is understood as shaped by interacting regulative (rules and sanctions), normative (field expectations and professional standards), and cultural-cognitive (taken-for-granted meanings) pillars; sustainability outcomes depend on the alignment of these pillars, not on policy adoption or resource inputs alone (Scott, 2014; Thornton et al., 2012). We define pillar misalignment as the co-occurrence of low sanction credibility (regulative), field norms that legitimate cost-minimizing labor practices and short time horizons (normative), and meaning systems that naturalize hardship or gendered role segregation (cultural-cognitive), observed consistently across interviews and site experiences (e.g., P1, P3, P5; Laundry Worker). Under such conditions, organizations are incentivized toward symbolic compliance displaying sustainability signals without transforming routines consistent with classic accounts of decoupling in low-monitoring environments (Meyer and Rowan, 1977; Bromley and Powell, 2012; Suchman, 1995).

### 5.1 The regulative pillar: weak enforcement and structural fragility

The first axis of institutional misalignment is rooted in the regulative pillar, specifically in labor conditions and wage governance. Labor conditions are the hinge linking institutional misalignment to stalled progress on decent work and poverty reduction. Participants repeatedly described wage compression, limited progression, and high churn as routine features of hotel employment, with supervisors noting predictable exits when new properties open or small wage differentials appear (Interview P1; Supervisor Quote). In institutional terms, formal rules without credible monitoring become “paper rules,” so organizations default to prevailing field norms that valorize flexibility over retention (North, 1990; Scott, 2014). This equilibrium erodes SDG 8.5 (full and productive employment with equal pay) and SDG 8.8 (safe working environments), and weakens SDG 1.2 (reduce poverty in all its dimensions) when wages fail to secure basic needs what hospitality scholars term working poverty (Baum et al., 2016b; Scheyvens and Biddulph, 2018). Evidence that unfulfilled pay and promotion promises trigger exit decisions “a lot of promises, but not fulfilled” further signals legitimacy loss and rational turnover under weak enforcement and low trust (Suchman, 1995; International Labour Organization (ILO), 2023a). International reporting shows that when labor inspection and wage-setting regimes lack bite, inflation and cost containment disproportionately harm low-wage service workers, amplifying precarity in sectors like hotels (International Labour Organization (ILO), 2023a,b). The decoupling of wages from the cost of living in Uzbekistan’s urban centers further threatens the achievement of SDG 10 (Reduced Inequalities). As Ahmad and Nisar (2022) point out, when the ‘fairness’ pillar of institutional theory is ignored, the resulting employee dissatisfaction creates a toxic organizational culture that becomes resistant to any future sustainability reforms.

### 5.2 The normative pillar: industry practices, cost-minimization, and the skills gap

A second axis of misalignment emerges from the normative pillar. The skills formation system provides a clear example of this. Respondents characterized tourism curricula as outdated “old Soviet standard program” and misaligned with operational realities, pushing firms to rely on tacit, on-the-job learning (Interviews P3, T1, HR4). This pattern aligns with institutional isomorphism: education providers reproduce legacy structures to maintain credibility with peers and regulators, stabilizing legitimacy while constraining responsiveness and innovation (DiMaggio and Powell, 1983; Scott, 2014). The result is symbolic proficiency credentials that signal readiness without conferring the capabilities hotels require necessitating costly retraining and fuelling frustration between “book knowledge” and workable practice (Meyer and Rowan, 1977; Bromley and Powell, 2012). Beyond service quality and productivity, this inhibits professional identity and mobility, holding back SDG 4.4 (skills for employment) and the upgrading central to SDG 8.2 (higher economic productivity via innovation) (Bramwell and Lane, 2011; Scott, 2014). Alignment therefore demands co-production of competency frameworks among universities, colleges, hotel associations, and regulators explicitly integrating sustainability, OSH, and gender-responsive HRD so that legitimacy accrues to providers who update capabilities rather than merely mirroring legacy templates (DiMaggio and Powell, 1983; Thornton et al., 2012).

Beyond skills formation, normative industry practices and cost-minimization are also deeply evident in infrastructural and occupational health deficits. Infrastructure emerges as the material residue of past institutional logics, locking firms into routines that are expensive to reverse even as norms shift toward greener, biophilic design. Participants depicted many hotels as dated, maintenance-heavy, and resource-intensive, with calls to “bring nature inside the hotels,” especially in urban properties (Interview P5). Such path dependence reflects prior investment rationalities and regulatory incentives emphasizing visible capacity and short-term returns over sustainability-led innovation (Scott, 2014; North, 1990). Managers may endorse sustainability yet face hard constraints embedded in assets, supplier contracts, financing, and staff skills (Greenwood et al., 2011; Thornton et al., 2012). Without enabling coercive and market mechanisms clear green-building codes, energy retrofit incentives, concessional finance, and training for engineering/housekeeping teams normative aspirations will not diffuse at scale, slowing progress on SDG 9.4 (upgrade infrastructure with resource efficiency and clean technologies) while forgoing co-benefits for worker well-being and guest experience (Bramwell and Lane, 2011; Scott, 2014).

Deficits in occupational health and safety reveal the sharpest gap between codified standards and operational practice. Back-of-house staff reported exposure to dust and chemicals without reliable PPE “we don’t have masks and gloves” indicating both weak enforcement and a normative tolerance of risk for lower-status roles (Laundry Worker; International Labour Organization (ILO), 2023a). In institutional terms, attention and resources concentrate on visible, guest-facing spaces, while hidden labor absorbs hazards as a taken-for-granted cost of doing business precisely the kind of asymmetric prioritization that sustains decoupling (Scott, 2014; Greenwood et al., 2011). These patterns violate SDG 3.9 (reduce illnesses from hazardous chemicals) and SDG 8.8, and they undermine organizational legitimacy when safety promises are experienced as symbolic (Suchman, 1995; Bromley and Powell, 2012). Restoring alignment requires predictable inspections, escalating sanctions for repeat violations, and embedding OSH metrics in audits, procurement, and performance systems so that sanctions and reputational rewards change the calculus of day-to-day decisions (North, 1990; Scott, 2014).

### 5.3 The cultural-cognitive pillar: gendered expectations and stigmatization

Finally, institutional misalignment is deeply anchored in the cultural-cognitive pillar. Gender inequality in hotels is driven less by capability than by deeply embedded cultural-cognitive schemas that naturalize women’s disproportionate unpaid care, stigmatize particular hotel roles, and devalue women’s career continuity. “Nobody thinks that I have a career worth keeping” even when formal openings exist (Women Respondents; Acker, 2006). Male narratives that hospitality work is “too tough” for women illustrate how meanings police the boundary of “appropriate” jobs, constraining participation and progression regardless of talent (Male Respondents; Scott, 2014). Because the cultural-cognitive pillar is sticky relative to formal rules, equal-opportunity policies alone yield partial or uneven gains unless care infrastructures, schedules, and promotion criteria are redesigned in tandem (Acker, 2006; Scheyvens and Biddulph, 2018). Advancing SDG 5.4 (recognize and value unpaid care) and SDG 5.5 (women’s participation and leadership) will hinge on institutional work that recodes what “good” hospitality work looks like: flexible scheduling without penalty, childcare support, anti-harassment enforcement, transparent competency-based progression, and visible female role models (Lawrence and Suddaby, 2006; Thornton et al., 2012).

Across domains, the regulative, normative, and cultural-cognitive pillars mutually reinforce one another: low sanction credibility pillars mutually reinforce one another: low sanction credibility enables cost-minimizing norms; those norms are stabilized by cultural beliefs that render hardship and churn “normal,” which in turn reduces pressure for enforcement. In such contexts, stand-alone programs, training workshops, CSR statements, or one-off upgrades tend to produce symbolic compliance rather than field-level transformation (Meyer and Rowan, 1977; Bromley and Powell, 2012). This helps explain why outcomes tied to SDG 1.2, 3.9, 4.4, 5.4/5.5, 8.5/8.8, 9.4 (and SDG 16.6 on effective, accountable institutions) remain stubbornly resistant to change even as sustainability rhetoric grows (Bramwell and Lane, 2011; Scott, 2014). Conceptually, the contribution is to show that tourism’s sustainability shortfalls are equilibrium properties of cross-pillar frictions, extending institutional explanations of decoupling in service sectors and clarifying why isolated initiatives underperform absent coordinated realignment (Greenwood et al., 2011; Thornton et al., 2012).

Alternative accounts resource scarcity, seasonality, or demographic labor supply undoubtedly shape cost containment and turnover. Yet the patterned persistence across firms and roles, the recurrent gap between formal rules and practice, and the normative acceptance of churn indicate a structural equilibrium better captured by institutional misalignment than by constraints alone (North, 1990; Meyer and Rowan, 1977). This boundary-conditions the claims while reinforcing the mechanism.

Practically, reform is sequence-sensitive. First, increase enforcement credibility: predictable inspections, transparent reporting, and escalating penalties for repeat non-compliance so that rules begin to shape daily choices (North, 1990; Scott, 2014). Second, recalibrate field norms through accreditation that ties legitimacy to training hours, OSH performance, and competency-based career ladders rewarding firms that invest in people and safety (DiMaggio and Powell, 1983; Suchman, 1995). Third, undertake institutional work on meanings campaigns and coalitions that normalize women’s advancement, revalue back-of-house labor, and showcase biophilic retrofits and ergonomic redesigns as markers of professionalism (Lawrence and Suddaby, 2006; Thornton et al., 2012). When rules bite, norms reward, and meanings legitimize inclusive, safe, and green operations, the sector can shift from symbolic adoption to routinized practice, improving retention by converting churn-accepting routines into a retention-positive equilibrium characterized by wage ladders, credible progression, safer work, and visible advancement pathways (Meyer and Rowan, 1977; Scott, 2014).

A key limitation of this study is that it draws on insights from fifteen participants across three hotels in Tashkent, making its scope context-specific. Although Tashkent reflects the most structured and regulated segment of Uzbekistan’s tourism sector, rural or less developed areas may encounter institutional voids in distinct ways. Accordingly, the results should be viewed as conceptually informative rather than statistically generalizable.

Moreover, the study centers solely on employee perspectives; viewpoints from managers, regulators, or policymakers could offer additional interpretations of sustainability shortcomings. Future studies might broaden the geographic scope, include a wider range of stakeholders, or adopt cross-sector comparative designs to further substantiate and extend these insights.

Despite these boundaries, the strong alignment of participant narratives across the three hotels and the coherence between the empirical evidence and institutional theory enhance the study’s credibility and support the transferability of its conclusions.

Overall, the findings suggest that institutional voids in Uzbekistan’s tourism sector are not isolated governance failures but structural misalignments across regulatory, normative, and cultural-cognitive pillars. The recurrence of similar patterns across multiple hotel contexts indicates that these dynamics reflect systemic conditions rather than firm-specific deficiencies. By tracing how weak enforcement, cost-minimizing industry norms, and gendered cultural expectations converge at the organizational level, this study demonstrates how macro-level institutional arrangements materialize in everyday workplace experiences. In doing so, it contributes to institutional theory by illustrating how sustainability gaps emerge through cross-pillar misalignment in emerging market contexts.

## 6 Significance of the study

### 6.1 Theoretical contribution

Hotel management must move beyond symbolic compliance by embedding sustainable HRM into daily operations. To combat turnover, hotels should formalize transparent, competency-based career progression ladders so employees see long-term value in their roles. Furthermore, to address gender inequality and cultural stigmas (SDG 5), hotels should implement targeted inclusive practices, such as hosting community career days or partnering with NGOs to create safe, subsidized transport options for female employees working late shifts. To accelerate this transition, domestic properties can participate in knowledge-sharing initiatives with international hotel chains operating in Uzbekistan to adopt established frameworks for inclusion and safety. Specifically, hotel management must focus on three practical areas: policy, by formalizing zero-tolerance anti-harassment rules and transparent, competency-based career ladders; checking, by instituting regular internal HR audits and anonymous employee feedback loops to monitor actual workplace conditions; and education, by providing continuous, modernized on-the-job training that not only bridges the gap left by outdated curricula but also sensitizes management to gender equity and occupational safety standards.

### 6.2 Contribution to practice

Hotel management must move beyond symbolic compliance by embedding sustainable HRM into daily operations. To combat turnover, hotels should formalize transparent, competency-based career progression ladders so employees see long-term value in their roles. Furthermore, to address gender inequality and cultural stigmas (SDG 5), hotels should implement targeted inclusive practices, such as hosting community career days or partnering with NGOs to create safe, subsidized transport options for female employees working late shifts.

### 6.3 Contribution to policy-making and local industry bodies

For workforce sustainability to transition from symbolic compliance to embedded practice, actionable interventions are required at both the governmental and industry levels. To strengthen the regulative pillar, the government must adopt a more proactive enforcement stance. Specifically, the Ministry of Employment and Poverty Reduction in collaboration with the Tourism Committee of the Republic of Uzbekistan should establish a dedicated hospitality labor task force responsible for conducting targeted, predictable audits of wage standards and occupational health and safety (OSH) compliance. Furthermore, the state should implement robust whistleblower protections by creating an anonymous, accessible grievance mechanism where hotel staff can report labor violations, unsafe chemical exposure, or withheld wages without fear of retaliation. Currently, specific whistleblower protection laws in Uzbekistan’s labor code remain underdeveloped for the service sector; enacting binding anti-retaliation legislation is a critical prerequisite for these mechanisms to function effectively.

To encourage proactive adoption, these regulatory measures should be balanced with incentives, such as tax benefits or accelerated licensing renewals for hotels that achieve recognized international certifications for fair labor practices and gender equity.

At the industry level, addressing normative and cultural-cognitive barriers requires coordinated action from local associations and hoteliers. To bridge the skills gap and directly support SDG 4, the Hoteliers Association of Uzbekistan, in partnership with universities, must co-produce an updated, practice-oriented apprenticeship model that transitions away from outdated legacy curricula. Moreover, industry bodies must actively work to destigmatize female hospitality employment through public awareness campaigns that showcase successful female leaders, thereby shifting entrenched cultural-cognitive perceptions. At the operational level, hotels must implement practical support systems, such as subsidized transport for late shifts and flexible scheduling, to accommodate women’s caretaking responsibilities. Finally, to ensure alignment with SDG 3, the sector should standardize OSH training by developing mandatory, cross-hotel training consortiums that provide back-of-house staff with certified training on handling chemicals and properly utilizing personal protective equipment.

## 7 Limitations and future research

A key limitation of this study is its cross-sectional nature; data was collected at a single point in time. Future longitudinal research could reveal how institutional voids evolve as Uzbekistan’s tourism policies mature. Second, the study focuses exclusively on 4-star hotels; 3-star and 5-star properties may experience different institutional pressures, with 5-star international chains potentially being more insulated from local voids. Third, given the cultural context, social desirability bias may have affected responses about sensitive topics, such as gender discrimination or safety violations, despite assurances of confidentiality. Finally, the study centers solely on employee perspectives in Tashkent; future studies might broaden the geographic scope to secondary cities and include a wider range of stakeholders, such as policymakers and hotel managers, to further substantiate these insights.

## 8 Conclusion

This study contributes to the literature on workforce sustainability by applying Institutional Theory to the rapidly expanding tourism sector of Uzbekistan. The qualitative findings suggest that the persistent gap between macro-level sustainability goals and frontline realities is not merely a symptom of managerial failure, but the result of cross-pillar institutional misalignments. Specifically, weak regulative enforcement, cost-minimizing normative practices, and cultural-cognitive gender expectations interact to produce an environment of symbolic compliance. For transition economies, achieving true workforce sustainability requires shifting from rapid infrastructural growth to intentional human capital development.

## Data Availability

The raw data supporting the conclusions of this article will be made available by the authors, without undue reservation.
